# Fetal asphyctic preconditioning alters the transcriptional response to perinatal asphyxia

**DOI:** 10.1186/1471-2202-15-67

**Published:** 2014-05-29

**Authors:** Kimberly EM Cox-Limpens, Johan SH Vles, Daniel LA van den Hove, Luc JI Zimmermann, Antonio WD Gavilanes

**Affiliations:** 1 School for Mental Health and Neuroscience (MHeNS), Maastricht University, Universiteitssingel 50, Room 1.152, Maastricht 6229 MD, The Netherlands; 2 Department of Pediatrics, Maastricht University Medical Center (MUMC), postbus 5800, Maastricht 6202, AZ, The Netherlands; 3 Department of Pediatric Neurology, Maastricht University Medical Center (MUMC), P.Debyelaan 25, Maastricht 6229, HX, The Netherlands; 4 Division of Molecular Psychiatry, Laboratory of Translational Neuroscience, Department of Psychiatry, Psychosomatics and Psychotherapy, University of Würzburg, Fuechsleinstrasse 15, Wuerzburg 97080, Germany; 5 Institute of Biomedicine, Faculty of Medicine, Catholic University of Guayaquil, Av. Carlos Julio Arosemena Km. 1 1/2 vía Daule, Guayaquil, Ecuador

**Keywords:** Perinatal Asphyxia, Fetal Preconditioning, Neonatal Brain,Neuroprotection, Microarray,Oxidative stress, Ubiquitination, Epigenetics

## Abstract

**Background:**

Genomic reprogramming is thought to be, at least in part, responsible for the protective effect of brain preconditioning. Unraveling mechanisms of this endogenous neuroprotection, activated by preconditioning, is an important step towards new clinical strategies for treating asphyctic neonates.

Therefore, we investigated whole-genome transcriptional changes in the brain of rats which underwent perinatal asphyxia (PA), and rats where PA was preceded by fetal asphyctic preconditioning (FAPA). Offspring were sacrificed 6 h and 96 h after birth, and whole-genome transcription was investigated using the Affymetrix Gene1.0ST chip. Microarray data were analyzed with the Bioconductor Limma package. In addition to univariate analysis, we performed Gene Set Enrichment Analysis (GSEA) in order to derive results with maximum biological relevance.

**Results:**

We observed minimal, 25% or less, overlap of differentially regulated transcripts across different experimental groups which leads us to conclude that the transcriptional phenotype of these groups is largely unique. In both the PA and FAPA group we observe an upregulation of transcripts involved in cellular stress. Contrastingly, transcripts with a function in the cell nucleus were mostly downregulated in PA animals, while we see considerable upregulation in the FAPA group. Furthermore, we observed that histone deacetylases (HDACs) are exclusively regulated in FAPA animals.

**Conclusions:**

This study is the first to investigate whole-genome transcription in the neonatal brain after PA alone, and after perinatal asphyxia preceded by preconditioning (FAPA). We describe several genes/pathways, such as ubiquitination and proteolysis, which were not previously linked to preconditioning-induced neuroprotection. Furthermore, we observed that the majority of upregulated genes in preconditioned animals have a function in the cell nucleus, including several epigenetic players such as HDACs, which suggests that epigenetic mechanisms are likely to play a role in preconditioning-induced neuroprotection.

## Background

Perinatal asphyxia (PA) is a major cause of neonatal mortality and is still the 2^nd^ largest cause of death among neonates worldwide [1]. Survivors often suffer permanent neurological deficits, such as motor disabilities, learning and cognitive problems [2]. The only evidence-based therapeutic strategy for treating term asphyxiated infants currently available is post-asphyctic hypothermia. However, only a subset of patients benefit from this strategy. Therefore, there is an urgent need to develop additional neuroprotective strategies that may, whether or not combined with hypothermia, provide an even better neurological outcome [3].

Several experimental pharmacologic therapeutic strategies were developed to prevent deleterious effects of perinatal asphyxia, such as the anti-oxidant allopurinol which should reduce free radical damage, and blockage of NMDA-receptors with magnesium which should prevent excitotoxicity. Although results in animal models seemed promising, clinical studies have not yet reached a favorable outcome with these treatments [4].

A different promising approach is studying endogenous brain protection provided by the physiological phenomenon of preconditioning. It was first described in the brain in 1964 with a report of prolonged survival in rats who underwent brief anoxia before a second anoxia period [5]. The primary sublethal hypoxic-ischemic event is called the preconditioning stimulus, and exposure to such a stimulus induces endogenous neuroprotective mechanisms [6]. However, the underlying mechanisms governing this phenomenon have not been fully elucidated yet. Insight into these mechanisms could provide us with directions for future neuroprotective strategies. It has been suggested that genomic reprogramming can explain a large part of these mechanisms and genome-wide microarray technology provides an excellent tool to investigate this neuroprotective reprogramming in experimental models [7].

So far, several studies have investigated large scale gene expression with microarray technology after preconditioning in the newborn or adult brain [8-11]. However, different experimental paradigms were used and, considering that no paradigm incorporated the fetal-to-neonatal transition, none of these truly resembles the global impact of PA and unique physiological mechanisms specific to the time of birth are missed.

Here we present a whole-genome microarray study in a previously validated model where we combine a global PA insult at the time of birth, with fetal asphyctic (FA) preconditioning on embryonic day 17 (E17). In this experimental model we have previously shown that for animals subjected to PA, FA preconditioning results in better survival, no abnormal apoptosis in the brain at P8, and no abnormal behavior in adults [12,13]. Furthermore, in a previous microarray study we were able to characterize whole-genome gene expression in FA animals, 96 hours after preconditioning which is just before birth [11]. Now, we aim to investigate the short- and long-term genomic response to PA, and perinatal asphyxia when preceded by fetal preconditioning (FAPA). We hypothesize that the FAPA animals show a neuroprotective gene expression pattern different from control and PA animals. Furthermore, we chose to take our microarray data-analysis beyond the single-gene approach, and subjected our data to pathway analysis in order to derive results with maximum biological relevance [14].

## Results

### Fetal preconditioning protects against abnormal apoptosis in DG

**Figure 1:**
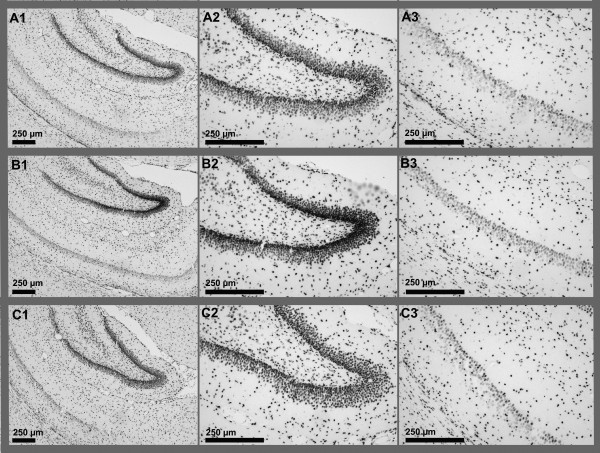
**Representative photomicrographs of Cleaved Caspase-3 staining. A1**-3: Control. **B1**-3: Perinatal asphyxia (PA) alone. **C1**-3: Fetal preconditioning and perinatal asphyxia (FAPA) combined. **A1**, **B1**, **C1**: overview at 4x magnification. **A2**, **B2**, **C2**: Dentate gyrus at 10x magnification. **A3**, **B3**, **C3**: **CA1** region at 10x magnification.

Figure 1 depicts representative photomicrographs of the Cleaved Caspase-3 staining. We observed no difference in activated Caspase-3 signal intensity the CA1 hippocampal region in P14 animals. However, in the dentate gyrus (DG) we observed a significant difference in activated Caspase-3 signal intensity with one-way ANOVA (p = 0.014), and post-hoc Bonferroni tests showed an increase of activated Caspase-3 signal intensity in the PA group compared to the control animals (111,5% compared to control, p < 0.05). No significant difference was observed for the preconditioned FAPA group, indicating functional fetal preconditioning.

### Whole genome expression profiling

**Figure 2:**
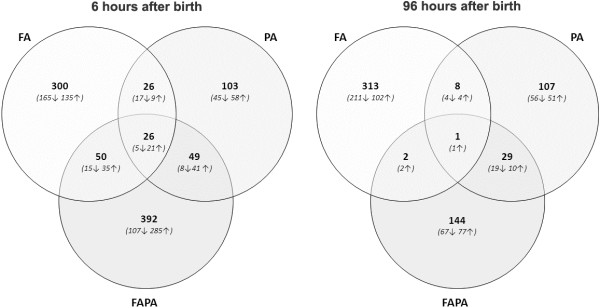
**Differential mRNA expression.** This Venn diagram displays the number of differentially expressed transcript per experimental group compared to controls, including the number of up- and downregulated transcripts, at 6 and 96 hours after birth. (FA: experimental group only subjected to fetal preconditioning. PA: experimental group only subjected to perinatal asphyxia. FAPA: experimental group subjected to both fetal preconditioning and perinatal asphyxia).

Whole-genome microarray technology was used to evaluate differential gene expression in animals that underwent FA, PA, or FAPA compared to controls, 6 and 96 hours after birth. Results are depicted in a Venn diagram in Figure 2. At 6 hours after birth we found the majority of differentially regulated genes in the FAPA group (517; 135↓ 382↑), then in the FA group (402; 202↓ 200↑), and finally in the PA group (204; 75↓ 129↑). At 96 hours after birth we see a different picture with the majority of differentially regulated genes in the FA group (324; 215↓ 109↑), then in the FAPA group (176; 86↓ 90↑), and finally in the PA group (145; 79↓ 66↑). The percentage of transcripts that are regulated in two (0.6-24%) or all three (0.3-25%) experimental groups is minimal, which leads us to conclude that the transcriptional phenotype of these groups is unique.

In order to derive the neuroprotective mechanisms that are active in the preconditioned brain and accomplish a better outcome when subjected to asphyxia, studying the effect of PA and FAPA will be most valuable. Therefore, we will focus our results on further evaluation of the PA and FAPA group.

### Effect of asphyxia on gene expression

**Figure 3:**
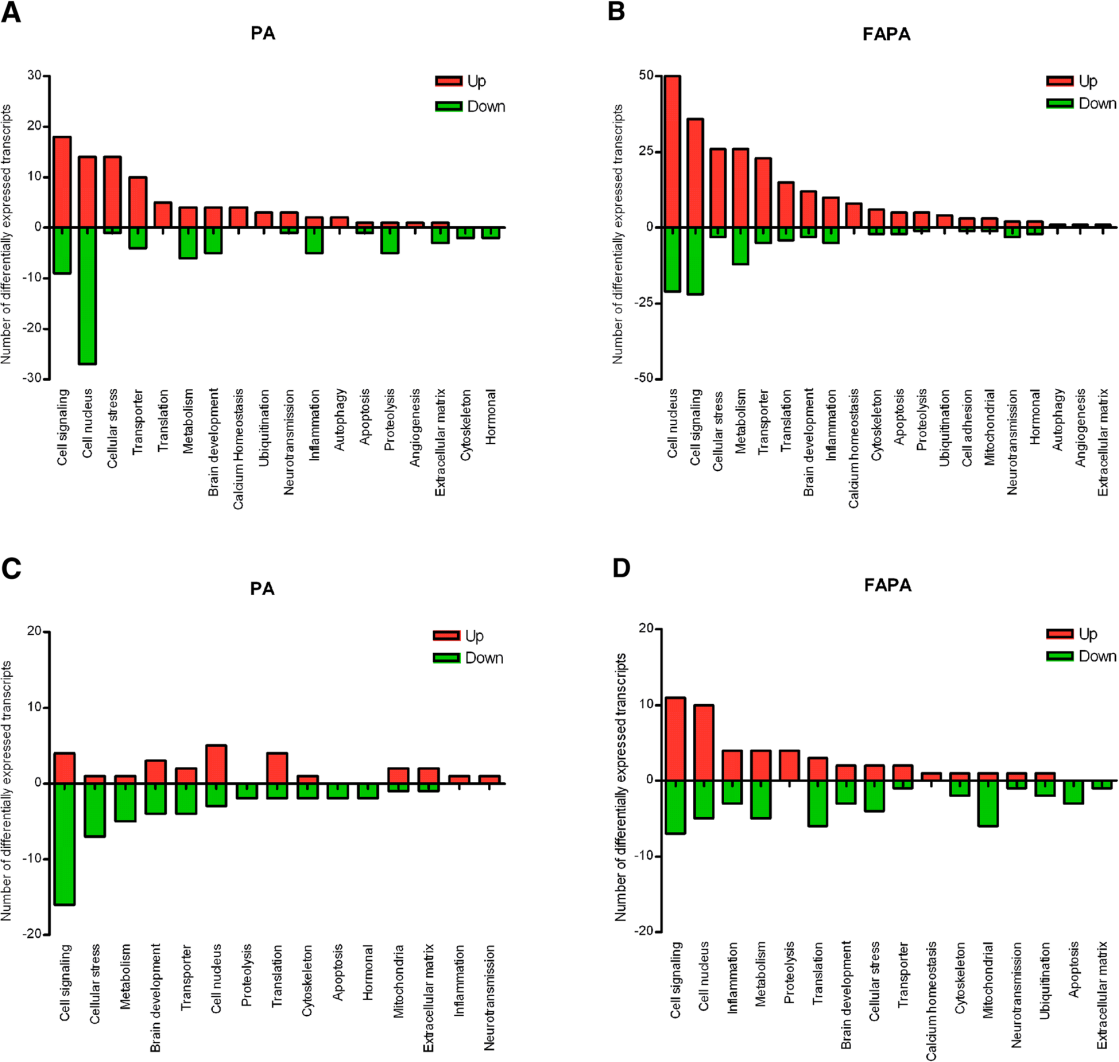
**Differential expression grouped according to biological function.** The number of upregulated transcripts is depicted by the red columns while the number of downregulated transcripts is depicted by the green columns. **A-B**: 6 hours after birth, PA and FAPA group respectively. **C-D**: 96 hours after birth PA and FAPA group respectively. (PA: experimental group only subjected to perinatal asphyxia. FAPA: experimental group subjected to both fetal preconditioning and perinatal asphyxia).

Gene expression analysis with the Bioconductor Limma package yielded 204 transcripts that were differentially expressed in PA animals compared to control 6 hours after perinatal asphyxia with a p-value <0.01, and fold changes ranging from 0.67 to 1.46 (complete list available in Additional file 1). We found 129 transcripts that were upregulated, and 75 transcripts that were downregulated 6 hours after PA.Differentially expressed transcripts were grouped according to their biological function (see Figure 3A). After asphyxia alone there is a marked upregulation of transcripts involved in the cellular stress response (Ddit3, Dnajb9, Hap1, Hspa13, Hspa5, Mt1a, Mt2a, RGD1561381, Sh3pxd2a, Txnl4b). Also, many upregulated transcripts are related to cell signaling pathways (Aktip, Dennd3, Ebpl, Galntl1, Gfra1, Gnb5, Gprasp2, Hcrtr2, Inpp1, Inpp5j, Limk2, Ppapdc1b, Rab22q, Rab24, Rab5a, Rasgef1c, Tmub2, V1re7). Furthermore, most differentially expressed transcripts have their function in the cell nucleus with the majority of these transcripts downregulated (Upregulated: Abt1, Jund, LOC100125368, Nap1l5, Pms2, Rbm11, Rexo4, Sfrs2ip, Swap70, Tspyl2, Zcchc12, Zfp385d, Znf763. Downregulated: Ccna2, Cenpe, Fancg, Helt, Hist2h2ac, Mcm4, Mzf1, Nfatc3, RGD1564126, Six2, Taf7l, Zdhhc25, Zfp36l3).

Among the genes that were exclusively regulated in the PA experimental group 6 hours after birth is the coiled-coil domain containing 8 (Ccdc8) which was downregulated after PA and known to be specifically required for p53-mediated apoptosis after DNA damage [15].

96 Hours after PA the number of differentially expressed transcripts was decreased to 145, with a p-value <0.01 and fold changes ranging from 0.72-1.59 (complete list available in Additional file 2). We found 66 transcripts that were upregulated, and 79 transcripts that were downregulated 96 hours after PA. Differentially expressed transcripts were grouped according to their biological function (see Figure 3C). The majority of upregulated transcripts has their function in the cell nucleus (Khdrbs2, Med1, Mllt3, Smarca2, Znf512), while the majority of downregulated transcripts is involved in cell signaling (Ak7, Evi2a, Gnal, Gng7, Gpr124, Itpka, Npy5r, Olr715, Olr880, Rab15, Rasgrp1, Rasip1, Rasl10b, Spa17, Sulf2, Taar7b) and cellular stress (Cant1, Cbs, Dnajc27, Mx1, Nkiras1, Nqo1, Parp12). Interestingly, among the genes related to cellular stress that were downregulated, 4 were exclusively regulated in the PA group (Cant1, Mx1, Nkiras1, Parp12). Moreover, the direction of transcriptional regulation 96 hours after PA is opposite to our findings 6 hours after PA for the following categories: “cell nucleus”, “cell signaling”, and “cellular stress”.

### Effect of asphyxia on gene expression in preconditioned animals

With whole-genome microarray 6 hours after PA we found 517 transcripts that were differentially expressed in FAPA animals compared to control, with a p-value < 0.01, and fold changes ranging from 0.62 to 1.95 (complete list available in Additional file 1). With 382 transcripts the majority of differentially expressed transcripts were upregulated. There were 135 downregulated transcripts in FAPA animals 6 hours after PA. An overview of differentially expressed transcripts grouped to biological function can be found in Figure 3B. The majority of upregulated transcripts have a function in the cell nucleus (Upregulated: Abt1, Adar, Arid5b, Atf4, Casc3, Cdkl1, Chd2, Cstf2t, Deadc1, Erg, Eya4, Gnl3, Hdac1, Hdac11, LOC100125368, Mbnl2, Mir29c, Morc2, Morg1, Nap1l5, Nr2c1, Prmt8, Prpf38b, Rfc2, Sfrs3, Taf1a, Tarsl2, Tspyl2, Tspyl5, Xbp1, yars, Zbtb25, Zbtb26, Zcchc12, Zfp110, Zfp143, Zfp235, Zfp385b, Zfp385d, Zfp397os, Zfp9, Zfr, Zmym1, Znf23, Znf507, Zswim3, Zwilch) or are involved in cell signaling (Upregulated: Acvr1b, Adap2, Adra1a, Aktip, Alkp1, Arfrp1, Arhgap10, Arl1, Arrdc4, Eltd1, Galnt11, Galnt14, Gfra1, Gprasp2, Grem1, Gtbpb8, Hcrtr1, Inpp1, Inpp5j, Olr1148, Olr121, Pde3a, Pde8b, Psd2, Rab24, Rab5a, Rasgef1c, Rasl11b, Rem2, Rerg, Rhoq, V1rf5, V1rg13, Vom2r66). Furthermore, we found that many upregulated transcripts play a role in cellular stress (Clec2g, Ddit3, Derl3, Dnajb3, Dnajb9, Gstm5, Hap1, Herpud1, Hif3a, Hspa13, Hspa5, Hspb1, Hyou1, LOC289614, Mgst2, Mt2a, Nfkbie, Nucb2, Ppp1r15b, Selk, Sels, Serp1, Tmx1, Txndc11, Txnl4b) and metabolism (Aadacl1, Abo, Acadm, Aldh18a1, Aldh1l2, Aloxe3, Ampd3, Asns, Aspa, B3galt5, Blvra, Comtd1, Cyp1b1, Fpgt, Gpd1, Gpt1, Mthfd2, Nus1, Osbp2, Pfkp, Phyhipl, Pla1a, RGD1565316, Sat1).

Among the transcripts that were exclusively regulated in FAPA animals 6 hours after PA there were many genes with their function in the cell nucleus. Interestingly, several of these are histone deacetylases (HDACs) which are enzymes known to remove acetyl groups from histone tails allowing the chromatin to wrap the DNA more tightly and thereby repress gene expression [16]. Another group of genes exclusively regulated in FAPA animals is related to the extracellular matrix and possibly the blood-brain barrier more specifically: integrins, neurexophilin and embigin.

96 Hours after PA the number of differentially expressed transcripts in preconditioned animals with a p-value <0.01 was decreased to 176, and fold changes ranging from 0.59 to 1.33 (complete list available in Additional file 2). There were 90 upregulated transcripts and 90 downregulated transcripts. An overview of differentially expressed transcripts grouped to biological function can be found in Figure 3D. The majority of differentially transcripts were involved in cell signaling (Upregulated: Gpr33, Gpr87, Gpr153, Gtpbp3, Olr522, Olr731, Olr1162, Olr1417, Olr1584, Olr1730, Tas2r124. Downregulated: Ebpl, Gnal, Gng7, Rgs18, Saps2, Tbc1d5, Tbc1d9b) or has a function in the cell nucleus (Upregulated: Brd9, Cep152, Exosc7, Hnrnpu, Hnrpd, Hoxc6, Lhx9, Nr0b2, Rnf213, Znf512. Downregulated: Cdyl, Gemin7l1, Kat2a, Sc65, Znf622). Among the genes exclusively regulated in FAPA animals are 6 transcripts related to mitochondrial function (Acadvl, Akr1c2, Atp5l, Chchd6, LOC691211, Ndufa2), which were all downregulated. Furthermore, we observed that at 96 hours after birth the transcripts with a function in ubiquitination pathway are exclusively regulated in FAPA animals.

### Validation of microarray results with RT-qPCR

Both on 6 hours and 96 hours after birth we randomly chose 4 transcripts with a significant result in microarray analysis for technical validation with RT-qPCR (see Table 1). We observed greater fold changes with RT-qPCR compared to microarray results which is a common finding [17].

**Table 1:** RT-qPCR validation of microarray findings

**Time-point after birth**	**Transcript**	**Experimental group**	**FC microarray**	**P-value microarray**	**FC**	**P-value**
					**RT-qPCR**	**RT-qPCR**
6 h	Fkbp14	FAPA vs PA	1.20	p < 0.01	1.34	p < 0.05
	Arid5b	FAPA vs C	1.22	p < 0.0001	1.87	p < 0.05
	Hspb1	FAPA vs PA	1.29	p < 0.001	1.46	p < 0.05
		FAPA vs C	1.19	p < 0.001	1.39	p > 0.05
	Cma1	PA vs C	0.82	p < 0.01	0.66	p < 0.05
		FAPA vs PA	1.20	p < 0.01	1.45	p > 0.05
96 h	Bloc1s2	FAPA vs C	0.78	p < 0.001	0.40	p < 0.01
	Rdh10	PA vs C	0.87	p < 0.001	0.63	p < 0.05
	Nkiras1	PA vs C	0.84	p < 0.001	0.66	p < 0.05
	Sc65	FAPA vs C	0.66	p < 0.01	0.46	p < 0.001
		FAPA vs PA	0.68	p < 0.01	0.65	p < 0.05

(PA: experimental group only subjected to perinatal asphyxia. FAPA: experimental group subjected to both fetal preconditioning and perinatal asphyxia).

At 6 hours after birth we observed upregulation of Fkbp14 in FAPA compared to PA with qPCR (p < 0.05) and similarly with microarray. An upregulation in FAPA compared to control was observed for Arid5b (p < 0.05) which is consistent with microarray findings. Similarly, upregulation in FAPA compared to PA was observed for Hspb1 (p < 0.05) which is consistent with microarray findings. Downregulation of PA compared to control and was found for Cma1 with qPCR (p < 0.05) similar to microarray results. The upregulation in FAPA compared to control for Hspb1, and upregulation in FAPA compared to PA for Cma1 could not be confirmed with qPCR.

At 96 hours after birth we observed downregulation in PA compared to control for Nkiras1 and Rdh10 with qPCR (p < 0.05) which is consistent with microarray results. Downregulation in FAPA compared to control was found for Bloc1s2 and Sc65 (p < 0.01, p < 0.001), similar to what we observed with the microarray. Furthermore, we confirmed downregulation of Sc65 in FAPA compared to PA with qPCR (p < 0.05).

### Changes in biological pathways: gene set enrichment analysis

In order to derive results with maximum biological relevance we decided to subject our data to a pathway based approach. The method we used was Gene Set Enrichment Analysis (GSEA) for which the entire data set was ranked according to moderate t-statistics.

At 6 hours after birth we observed significant enrichment of 6 gene sets which can be found in Table 2. The KEGG pathway ‘protein processing in endoplasmatic reticulum’ was upregulated in both PA and FAPA animals compared to control. Another gene set related to the endoplasmatic reticulum (ER) is the KEGG pathway ‘N-glycan biosynthesis’ which is only upregulated in the FAPA group. N-glycans are very important for proper protein folding in the ER, they have binding residues for chaperone proteins, and they are known to be upregulated in the unfolded protein response [18]. This unfolded protein response has also been demonstrated shortly after transient global ischemia [19]. Furthermore, we observed upregulation of the KEGG pathway ‘aminoacyl-tRNA biosynthesis’ in the FAPA group. Similarly, a study of ischemia preconditioning in the rat retina found upregulation of genes with aminoacyl-tRNA synthetase activity [20]. Finally, in FAPA animals we observed upregulation of the Krüppel associated box (KRABbox) domain which is present in many zinc finger transcription factors and has been shown to be effective repressors of transcription [21]. One gene set is downregulated 6 h after birth in FAPA and this is the KEGG pathway ‘steroid biosynthesis’. Interestingly, steroid administration has previously been shown to aggravate neonatal hypoxic-ischemic brain injury [22]. Similarly, a drug that suppresses steroid biosynthesis was shown to prevent excitotoxic injury in cortical neurons [23].

**Table 2:** Gene Set Enrichment Analysis (GSEA) results

**Time of sacrifice**	**Gene set name**	**Description**	**NES**	**FDR q-value**	**Direction**	**Experimental group**
6 h	KEGG:04141	Protein processing in endoplasmatic reticulum	2.54	<0.001	↑	PA and FAPA
	PF01352	KRABbox	2.42	<0.001	↑	FAPA
	PF00956	Nucleosome assembly protein	2.14	<0.01	↑	
	KEGG:00970	Aminoacyl-tRNA biosynthesis	2.47	<0.001	↑	
	KEGG:00510	N-glycan biosynthesis	2.04	<0.01	↑	
	KEGG:00100	Steroid biosynthesis	-2.58	<0.001	↓	
96 h	GO:0005746	Mitochondrial respiratory chain	-2.20	<0.01	↓	PA and FAPA
	KEGG:00190	Oxidative phosphorylation	-2.34	<0.001	↓	
	GO:0001505	Regulation of neurotransmitter levels	-2.22	<0.01	↓	PA
	GO:0007268	Synaptic transmission	-2.11	<0.01	↓	
	GO:0007269	Neurotransmitter secretion	-2.23	<0.01	↓	
	KEGG:04020	Calcium signaling pathway	-1.97	<0.01	↓	
	KEGG:04970	Salivary secretion	-2.16	<0.01	↓	
	KEGG:04971	Purine metabolism	-2.04	<0.01	↓	
	PF00089	Trypsin	2.23	<0.01	↑	FAPA
	GO:0019208	Phosphatase regulator activity	-2.09	<0.01	↓	
	KEGG:03010	Ribosome	-2.237	<0.001	↓	
	BIOCARTA_PGC1APATHWAY		-2.09	<0.01	↓	

Significantly enriched gene sets found with GSEA using the FDR q-value <0.01 as cut-off for significance. NES = Normalized Enrichment Score. FDR q-value = False Discovery Rate method of correction for multiple testing. (PA: experimental group only subjected to perinatal asphyxia. FAPA: experimental group subjected to both fetal preconditioning and perinatal asphyxia).

At 96 hours after birth we found 12 gene sets that were significantly regulated in one or both experimental groups when compared to controls. Two gene sets involved in mitochondrial respiration, and more specifically the electron transport chain, were downregulated in both PA and FAPA animals. When looking at the leading edge analysis of these gene sets we observed that this downregulation mainly involves mitochondrial complex I (NADH dehydrogenase [ubiquinone]) and IV (cytochrome c oxidase) specific enzymes. This inhibition of mitochondrial complex I and IV, irrespective of ischemic preconditioning, was previously shown in global ischemia in adult rats [24]. Furthermore, we also observed genes involved in mitochrondrial complex II (succinate dehydrogenase), mitochondrial complex III (ubiquinol cytochrome c reductase) and mitochondrial complex V (ATP synthase) within both of the downregulated gene sets. Downregulation of these mitochondrial respiratory complexes can be beneficial following cerebral hypoxia-ischemia because they are the main source of Reactive Oxygen Species (ROS) in reperfusion [25]. Only in the PA group we observed downregulation of three GO terms related to neurotransmitters and synaptic transmission. Furthermore, the KEGG pathways ‘Calcium signaling pathway’, ‘Salivary secretion’, and ‘Purine metabolism’ are exclusively downregulated in PA animals and contain genes that have a function in cellular signaling such as adenylate cyclase, calmodulin, calcium/calmodulin-dependent protein kinase, ATPase, protein kinase A, and protein kinase C.

Exclusively in FAPA animals we observed a downregulation of the KEGG pathway ‘Ribosome’ which includes many ribosomal proteins and an ubiquitin residue. A previous study observed abnormal protein aggregation in transient cerebral ischemia resulting in irreversible destruction of protein synthesis machinery. Interestingly, this abnormal protein aggregation was prevented in preconditioned animals, and the downregulation of ribosomal gene expression we observed here could explain this phenomenon [26]. Finally, exclusively in FAPA we observed upregulation of the ‘Trypsin’ gene set which includes many different proteases, mainly serine proteases.

## Discussion

Here we present the first whole-genome expression data in neonatal brain tissue after PA and PA preceded by fetal preconditioning (FAPA). Following PA, we observed a gene expression pattern in preconditioned animals that differs significantly from control and PA animals.

### From univariate to pathway analysis

We chose to take our microarray data analysis beyond the single-gene approach in order to derive results with maximum biological relevance. Analyzing microarray results is typically done by comparing genes on a gene-by-gene basis and assessing if these genes are differentially expressed between experimental groups. Using this univariate approach the focus is on the genes that show the largest difference in expression between the experimental groups. However, there are some vital limitations to this approach. Most importantly univariate analysis assumes that all genes act independently of one another, and this is not the case in cell biology. It is well known that biological processes often affect sets of genes that act simultaneously. Therefore, a small increase in all genes that belong to a certain pathway is likely to be more biologically relevant then a high increase in a single gene in that pathway [27]. In addition, with pathway analysis added to the standard analysis of microarray data it is likely that we will find better overlap in results when different studies are investigating the same model [14]. There are many different methods available to conduct pathway analysis with microarray data. We chose Gene Set Enrichment Analysis (GSEA) in favor of a Singular Enrichment Analysis (SEA), such as DAVID, because GSEA does not require any pre-selection of genes and thereby avoids arbitrary factors needed for pre-selection. Moreover, because GSEA uses all information obtained in a microarray experiment it is best suited to allow genes with minimal changes to contribute to the enrichment analysis [27].

Interestingly, GSEA results revealed a similar, but more extensive perspective than our Limma analysis results. Both indicate that the most upregulated genes were involved in processes within the cell nucleus, and that the most downregulated genes were involved in signal transduction and synaptic transmission. However, GSEA provided more information on the pathways that are involved.

### Differential mRNA expression

When comparing the number of differentially expressed transcripts among the different experimental groups we notice that the FA group has the largest number of differentially expressed transcripts. This was surprising since in previous studies, where we performed behavioral tests at 6 months of age, FA animals score similar to control [12]. However, a study of focal ischemic preconditioning in adult mice had similar results with the largest number of differentially regulated transcript in the preconditioning only group [8].

For a further description of the data we chose to focus on the PA and FAPA experimental groups because both underwent the injurious PA and our aim was to show how the FAPA animals differ in their response to hypoxia-ischemia in order to derive the neuroprotective mechanisms that are in place after fetal preconditioning. The differential gene expression pattern in FAPA animals is a result of preconditioning-induced gene expression and PA-induced gene expression; moreover, preconditioning can also prevent the upregulation of certain PA-induced genes.

Interestingly, when we look at our results 96 hours after birth we observe that still over a hundred transcripts are differentially expressed in both PA and FAPA groups. This suggests that the transcriptional response to PA and fetal preconditioning is a durable one.

Finally, we need to be aware that it is mRNA expression we are investigating and although this can give great insight into cellular biology, gene expression does not necessarily translate into protein expression. We need to consider the facts that ischemia can both decrease protein production [28], and that hypoxic conditions can increase the stability of mRNA for specific transcripts which could lead to false positives [29].

### Cellular stress response is different in preconditioned animals

Interestingly, when comparing genes that are exclusively expressed in respectively the PA and FAPA groups 6 hours after birth we observe that FAPA animals show clearly more differentially expressed transcripts (29 vs 15) related to cellular stress. Exclusively in PA animals we observe an upregulation of several genes known to be induced by oxidative stress such as coiled-coil domain-containing 8 (Ccdc8) which plays a role in p-53 mediated apoptosis after DNA damage, and sequestosome 1 (Sqstm1) which is an ubiquitin binding protein involved in oxidative stress and autophagy. In both groups we see upregulation of metallothionein 2a (Mt2a) which is thought to protect cytosolic creatine kinases against stress by oxidants [30]. Although we see an upregulation of transcripts related to the antioxidant thioredoxin (Txndc11, Txnl4b) in both groups, the activation of antioxidants is clearly more pronounced in the FAPA group. We observed upregulation of antioxidants such as selenoproteins, glutathione S-transferase, and thioredoxin-related transmembrane protein (Tmx1, Selk, Sels, Mgst2, Gstm5) in FAPA animals 6 hours after birth. Moreover, we observe an upregulation of NF-kappaB inhibitor epsilon (Nfkbie) in FAPA animals. Nfkbie is known to trap Nfkb in the cytoplasm to prevent it from activating genes in the nucleus [31]. Activation of the alpha subtype of NF-kappaB inhibitors has been proposed to be modulated by nitric oxide and was shown reduce neuronal damage following focal cerebral ischemia [32]. Similarly, treatment with palmitoylethanolamide in transient ischemia was shown to inhibit degradation of the alpha subtype of NF-kappaB inhibitors and convey neuroprotection [33]. Although the epsilon subtype of NF-kappaB inhibitors (Nfkbie) has not been studied in cerebral ischemia, our data together with previous studies for different inhibitor of NF-kappaB subtypes suggest it could be a promising target for further study.

### The importance of ubiquitination and proteolysis

At 6 h after birth we see an upregulation of 2 transcripts related to ubiquitination in both groups, but these are not the same. In the PA group we observe upregulation of Fbxo6 and Psma3, while in FAPA animals we observe upregulation of Fbxo3 and Fbxo32. These transcripts mainly belong to the F-box family which is a crucial component of the Skp, Cullin, F-box containing complex (SCF complex) which besides it role in ubiquitination has an important role in cell cycle progression. Interestingly, at 96 h after birth genes related to ubiquitination are exclusively regulated in FAPA group (Uqcrq, and March2 down, Ube2q2l up). There is increasing evidence for the crucial importance of ubiquitination and subsequent proteolysis in oxidative stress and ischemia [34]. Still, experimental evidence can seem contradicting when in a model of isoflurane preconditioning neuroprotection is accompanied by attenuation of ubiquitin-conjugated protein aggregates [35], and in a different study intravenous treatment with ubiquitin was found to be neuroprotective [36]. Congruently with the upregulation of ubiquitin related transcripts in the FAPA group, we observed upregulated of transcripts involved in proteolysis and similarly with GSEA we observed significant upregulation of a gene set (PF: Trypsin) that comprises many proteases. Proteases are the effectors of proteolysis and therefore protein degradation. Ischemia leads to ER stress and protein misfolding. Furthermore ischemia is known to damage protein degradation pathways which altogether results in aggregation of unfolded proteins, translational arrest and ultimately cell death. Our findings of upregulated proteases in the preconditioned group are consistent with findings in ischemic postconditioning where an increased proteasome activity was found, as well as the observation that ischemic tolerance is blocked by proteasome inhibitors [34].

### Involvement of epigenetic mechanisms

We observed a striking number of differentially regulated transcripts with a function in the cell nucleus. The majority of transcripts related to the cell nucleus are regulated 6 hours after birth in FAPA. Here, we observe an upregulation of 50 transcripts, while 21 are downregulated. Many of these belong to the zinc finger family of transcription factors which were once considered function exclusively as sequence-specific DNA-binding motifs but are increasingly known for other functions such as their protein binding abilities [37]. The induction of zinc fingers in cerebral ischemia has been previously described but unfortunately their role in ischemia or neuroprotection has not been studied [38]. Besides differential regulation of zinc fingers we observe differential expression of several histone deacetylases (Hdac1, Hdac10, and Hdac11) exclusively in the preconditioned FAPA group. HDACs are enzymes that have the ability to remove acetyl groups from histones thereby repressing gene expression [39]. This suggests that there might be epigenetic mechanisms involved in preconditioning-induced neuroprotection, which is consistent with our findings in prenatal brain 96 hours after fetal preconditioning alone [11]. Similarly, in a recent review epigenetic changes were suggested to be the ‘master switch’ for activating neuroprotective pathways after preconditioning [40]. Even though there is growing evidence for a role of epigenetic mechanisms in neuroprotection, the evidence today is contradictory regarding the mechanisms of action. For example, different types of HDAC family members seem to exert different functions in mediating neuroprotection, and may cause different responses in different cell types. Additionally, we need to keep in mind that besides their ability to modulate chromatin, HDACs can also exhibit non-histone effects such as acetylation of transcription factors [41]. Furthermore, besides the ability to modify proteins, such as histones and transcription factors, it is important to realize that HDACs themselves can be modified posttranslationally [16].

Another finding that indicates the involvement of epigenetic mechanisms is the upregulation of the KRAB-box protein domain which is found with GSEA exclusively in FAPA animals 6 hours after birth (see Table 2). We have already mentioned how the KRAB domain is an effective gene repressor but it is the ability to induce reversible heterochromatization which clearly links it to epigenetic mechanisms. Similarly, the KRAB domain has been shown to induce de novo promoter methylation, albeit irreversible gene silencing, when generated in the first few days of mouse development [42].

Altogether, our results lend support for more in-depth research of epigenetic mechanisms involved in neuroprotection.

## Conclusions

This study is the first to investigate whole-genome transcription in the neonatal brain after PA alone and when preceded by fetal asphyctic preconditioning.

After perinatal asphyxia alone we found that the majority of differently regulated transcripts were upregulated in the early phase. A great deal of these upregulated transcripts is involved in the cellular stress response and cell signaling, which makes our findings consistent previous microarray research where different model approaches were used, and at the same time lends validity to previous findings . In addition we describe several genes and pathways, such as ubiquitination and proteolysis, which were not previously linked to preconditioning and/or neuroprotection.

Finally, we found that the majority of upregulated genes in preconditioned animals have a function in the cell nucleus, including several epigenetic players such as HDACs, which suggests that epigenetic mechanisms play a role in preconditioning-induced neuroprotection.

## Methods

### Animal model

All experiment protocols were approved by the Animal Ethics Board of Maastricht University according to Dutch governmental regulations. Adult Sprague-Dawley rats (body weight 250-320 g), obtained from Charles River (France), were kept under standard laboratory conditions with food and water given ad libitum, 21 ± 2˚C environment temperature, a 12 h light/dark schedule (lights on at 07:00 h) and background noise provided by radio. Breeding and fetal asphyctic (FA) preconditioning was carried out as previously described [11]. In brief, FA preconditioning was induced on E17 by completely clamping both uterine and both ovarian arteries with removable clamps for 30 minutes. Control dams were left undisturbed until day E21. On E21 all pregnant dams were observed for signs of labor, and after vaginal delivery of the first pup the mother was decapitated and the uterus was removed by Caesarean section. For PA and FAPA offspring, the uterus still containing the unborn pups was immediately placed in a water bath at 37°C for exactly 19 minutes to induce PA. Subsequently, pups were removed from the uterine horns, cleaned with gauze swabs and stimulated to breath inside a pediatric incubator (37°C, 60-80% humidity, room air). Finally, pups were randomly cross-fostered to surrogate dams. In this study we used male offspring exclusively because both morphological and behavioral evidence show a differential vulnerability to a birth insult in males versus females. A greater impact is seen in the male gender, possibly due to the difference in circulating sex hormones compared to females [43].

### Tissue preparation

**Figure 4:**
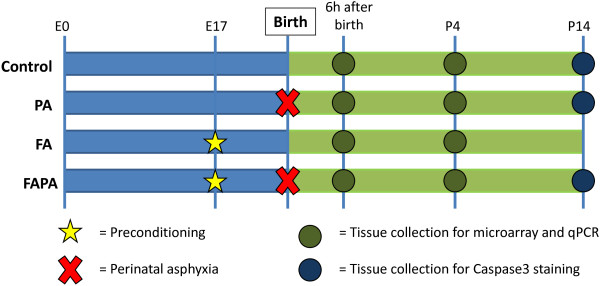
**Experimental design.** (FA: experimental group only subjected to fetal preconditioning. PA: experimental group only subjected to perinatal asphyxia. FAPA: experimental group subjected to both fetal preconditioning and perinatal asphyxia).

An overview of the experimental design is depicted in Figure 4. Pups were sacrificed by decapitation at two different time-points: 6 and 96 hours after birth. We chose these time-points in order to investigate the early and late transcriptional response respectively.

For microarray analysis, left hemispheres were dissected and submerged in RNA stabilizing reagent (Qiagen Benelux BV, Venlo, The Netherlands) before being snap-frozen in liquid nitrogen, and ultimately stored at -80°C. To prevent litter effects only 1 pup per dam was used for microarray analysis, with n = 5 per condition.

For RT-qPCR analysis right hemispheres were dissected, snap-frozen in liquid nitrogen, and ultimately stored at -80°C. A maximum of two pups per dam were used for and RT-qPCR (n = 5-8).

### Assessment of apoptosis

Complete brains of P14 animals were dissected and fixated by immersion in paraformaldehyde-based fixative containing glutaraldehyde (20% paraformaldehyde, 25% glutaraldehyde in 0.2 M phosphate buffer containing 15% picric acid) for 2 hours, before immersion in paraformaldehyde-based fixative without glutaraldehyde for 10 days. After immersion in 10% sucrose for 1 day, and 4 days in 20% sucrose, brains were frozen with CO2 and stored at -80°C. Frozen coronal sections (16 μm) were cut on glass slides using a cryostate. After postfixation of 10 minutes in 4% paraformaldehyde, sections were quenched in TBS containing 3% hydrogen peroxide for 20 minutes, then incubated in TBS containing 3% donkey serum, followed by overnight incubation with anti-Cleaved Caspase 3 (Asp175, 1:5000, Cell Signalling Technology UK). The next day, sections were incubated with secondary antibody for 2 hours (donkey anti-rabbit biotinylated, 1:200, Jackson ImmunoResearch USA). Finally, sections were incubated with avidin biotin complex (ABC reagent, 1:100, Vector Labs UK) for 1 hour before staining was revealed using 3,3′diaminobenzidine (DAB, Vector Labs UK) for 10 minutes. Using an AX70 microscope (Olympus NL), photomicrographs were taken at 4x and 10x magnification with Olympus Cell^P software version 5.1. Per animal, 3-5 sections were delineated between Bregma -3.14 and -4.30, and for each delineation, the mean gray value was calculated in ImageJ (version 1.45 s, NIH USA). Immunohistochemical staining, delineation and gray value calculation were performed in a blind fashion.

### Microarray analysis

For microarray analysis total RNA extraction and purification were performed on mini RNeasy columns (Qiagen Benelux BV, Venlo, The Netherlands), according to the manufacturer’s instructions. Quantity and purity of total RNA was determined by spectrophotometer analysis using the Nanodrop ND-1000 (Thermo Fisher Scientific Inc., Waltham, USA). Only samples with a 260/280 ratio between 1.8 and 2.1, and a 260/230 ratio between 1.5 and 2.0 were selected for microarray analysis. Additionally, RNA quality measurements were performed with Bioanalyzer 2100 (Agilent Technologies Netherlands B.V.). Samples with an RNA integrity number (RIN) below 8 were excluded.

Using the Ambion WT Expression Kit, per sample, an amount of 100 ng of total RNA spiked with bacterial poly-A RNA positive controls (Affymetrix Inc., Santa Clara, USA) was converted to double stranded cDNA in a reverse transcription reaction. Next, the sample was converted and amplified to antisense cRNA in an in vitro transcription reaction which was subsequently converted to single stranded sense cDNA. Finally, samples were fragmented and labeled with biotin in a terminal labeling reaction according to the Affymetrix WT Terminal Labeling Kit. A mixture of fragmented biotinylated cDNA and hybridization controls (Affymetrix Inc., Santa Clara, USA) was hybridized on Affymetrix GeneChip Rat Gene 1.0 ST Arrays followed by staining and washing in a GeneChip® fluidics station 450 (Affymetrix Inc., Santa Clara, USA) according to the manufacturer’s procedures. To assess the raw probe signal intensities, chips were scanned using a GeneChip® scanner 3000 (Affymetrix Inc., Santa Clara, USA). According to MIAME requirements data were submitted the NCBI GEO database, and are available under accession number: GSE42676.

### RT-qPCR

Total RNA was extracted with Trizol® reagent (Invitrogen, Paisley Scotland, UK) according to manufacturer’s instructions. Next, cDNA was generated with RevertAid First Strand cDNA synthesis kit (Fermentas GMBH, St. Leon-Rot, Germany). RT-qPCR reactions were carried out using SYBR green PCR master mix and the LightCycler 480 (Roche Diagnostics, Almere, The Netherlands). All primers were designed using Primer-BLAST. To control for error between samples we used the geometric mean of Ywhaz, Rpl13a, ß-actin and Hprt1 for normalization. We selected these reference genes based on their stability across our experimental conditions. Sequences of all primers used can be found in Table 3.

**Table 3:** Primer design for RT-qPCR

**Transcript name:**	**Forward:**	**Reverse:**
HPRT1	5′-AGACGTTCTAGTCCTGTGGC	5′-TGCAAATCAAAAGGGACGCA
β-actin	5′-GCCTTCCTTCCTGGGTATG	5′-GGTCTTTACGGATGTCAACG
Ywhaz	5′- AGACAGCACGCTAATAATGC	5′- CAGACAAAGGTTGGAAGGC
Rpl13a	5′- CCCTCCACCCTATGACAAG	5′- AGGTAAGCAAACTTTCTGGTAG
Fkbp14	5′- ACGACGACTGGAAACTCTCT	5′- CTGCTTCCCTACAGTTCGTC
Hspb1	5′- GGATCGATGACATGAGCAGC	5′- TGCCAGTAGCCTTCAACTCT
Arid5b	5′- CCGAGACTGTCATCCCAAAG	5′- TTCCGGAAGTTCTCCAGTCT
Cma1	5′- ACCAAAGCTGGGGAGATCAT	5′- ACTGCAGGCTGACAGGTAAT
Sc65	5′- AGGCGAACCGACTAGAGAAG	5′- AAAGTCCCCGCTGTTGTAGA
Rdh10	5′- TCCTGGTCAATAACGCTGGT	5′- TAGTGGTCCAGAAGTGTGCG
Bloc1s2	5′- AACTGACAGCCACCAGTGAA	5′- CCTGCTCCTCAATCACGTTG

### Gene Set Enrichment Analysis (GSEA)

For GSEA, a total of 737 rattus norvegicus gene sets were assembled, including 196 KEGG pathways (release 59.0), 81 Biocarta pathways (accessed August 18^th^ 2011), 184 Gene Ontology terms (AmiGO version 1.8), and 276 Pfam protein families database (Pfam 25.0). Each gene-set contained a minimum of 15 genes and a maximum of 500 genes in accordance with GSEA recommendations. The analysis was conducted using the GSEA software v2.07, provided by the Broad Institute (Cambridge, MA, USA) [14]. Ultimately, we performed a ‘Leading Edge Analysis’ in GSEA on significantly enriched gene-sets, to identify the genes that contribute most to the enrichment signal.

### Statistics

Analysis of the microarray data was performed in the R programming environment (version 2.12.0), with the packages developed within the Bioconductor project. The analysis was based on the RMA expression levels of the probe sets. Differential expression was assessed with the Limma package using moderated t-statistics. Results are presented as fold changes compared to control, and p-values < 0.01 were considered statistically significant.

For RT-qPCR, all data were distributed normally as tested with Kolmogorov-Smirnov test. Statistical significance was tested with the unpaired, two-tailed Student’s t-test. Results are presented fold change, means + SEM, and p-values <0.05 were considered statistically significant.

For GSEA, the microarray dataset was pre-ranked using moderated t-statistics. A gene set enrichment score (ES) was calculated based on Kolmogorov-Smirnov statistics and for each gene set the ES was normalized to account for difference in gene set size. Finally, a false discovery rate (FDR) was calculated relative to the normalized enrichment score (NES) values to determine the probability of type I errors. To control for multiple testing we used the false discovery rate (FDR) as described by Benjamin and Hochberg. Enriched gene-sets with an FDR q-value <0.01 were selected.

## Abbreviations

FA: Fetal Asphyctic preconditioning; PA: Perinatal Asphyxia; FAPA: Perinatal Asphyxia preceded by Fetal Asphyctic preconditioning; GSEA: Gene Set Enrichment Analysis.

## Competing interests

The authors declare that they have no competing interests (both financial and non-financial).

## Authors’ contributions

KC participated in the design of the study and performed the animal experiments, RNA extractions, qRT-PCR experiments, and immunohistochemical stainings. JV, DH and LZ participated in the design of the study and helped to draft the manuscript. AG conceived of the study and helped to draft the manuscript. All authors read and approved the final manuscript.

## Supplementary Material

Additional file 1 Complete list of differentially expressed transcripts 6 hours after birth in PA and FAPA animals compared to controls. Click here for file

Additional file 2 Complete list of differentially expressed transcripts 96 hours after birth in PA and FAPA animals compared to controls. Click here for file
